# EGFR-Ras-Raf Signaling in Epidermal Stem Cells: Roles in Hair Follicle Development, Regeneration, Tissue Remodeling and Epidermal Cancers

**DOI:** 10.3390/ijms141019361

**Published:** 2013-09-25

**Authors:** Eszter Doma, Christian Rupp, Manuela Baccarini

**Affiliations:** Max. F. Perutz Laboratories, Department of Microbiology, Immunology and Genetics, University of Vienna, Doktor-Bohr-Gasse 9, 1030 Vienna, Austria; E-Mails: eszter.doma@univie.ac.at (E.D.); c.rupp@univie.ac.at (C.R.)

**Keywords:** epidermis, hair follicle, stem cell, EGFR signaling, Ras, Raf, SSC, BCC, wound healing

## Abstract

The mammalian skin is the largest organ of the body and its outermost layer, the epidermis, undergoes dynamic lifetime renewal through the activity of somatic stem cell populations. The EGFR-Ras-Raf pathway has a well-described role in skin development and tumor formation. While research mainly focuses on its role in cutaneous tumor initiation and maintenance, much less is known about Ras signaling in the epidermal stem cells, which are the main targets of skin carcinogenesis. In this review, we briefly discuss the properties of the epidermal stem cells and review the role of EGFR-Ras-Raf signaling in keratinocyte stem cells during homeostatic and pathological conditions.

## 1. Introduction

The stratified, multilayered epidermis is the outermost barrier of the skin that protects the body from the environment. To maintain epidermal integrity, terminally differentiated cells at the surface of the interfollicular epidermis (IFE) are continuously replaced by proliferative cells of the basal layer [1].

By contrast, the hair follicle undergoes cyclic periods of growth (anagen) followed by regression (catagen) and rest (telogen) [2]. The constant renewal of the IFE and the rhythmic regeneration of the hair follicle governed by stem cells make the epidermis a powerful model for studying the behavior and signaling of adult stem cells in homeostatic and pathologic conditions [3].

This review provides an overview of the characteristics of hair follicle stem cells (HFSC) and IFE progenitor cells and of the role of the EGFR-Ras-Raf signaling pathway during normal skin maintenance, epidermis regeneration and carcinogenesis.

## 2. Epidermal Stem Cells

### 2.1. Characteristics

Epidermal stem cells are multipotent, slow-cycling cells with high proliferative potential, self-renewal capacity, and the ability to divide asymmetrically, giving rise to self-renewing and differentiating daughter cells [4,5]. In this way, stem cells supply short-lived, fast dividing and more differentiated cells, the so-called transit amplifying (TA) cells, which have a more reduced self-renewal capacity [4]. They are responsible for tissue growth and, after a number of cell divisions, give rise to terminally differentiated populations.

In addition to epidermal stem cells, recent studies have identified another cell type which might have an important role in tissue maintenance: the committed progenitor (CP) cells [6–9]. These cells do not originate from slow-cycling stem cells but rather exist in parallel, continually divide a limited number of times and differentiate stochastically. However, the exact definition of CP cells is yet unclear, and the concept is still evolving [10].

### 2.2. The Stem Cell Niche

Epidermal stem cells are located in niches, specific microenvironments that control their cycling behavior and maintain their undifferentiated state.

In mammals, hairy skin is built up from pilosebaceous units, composed of hair follicles and the surrounding IFE, which contain different types of stem cells. Based on the columnar appearance of the suprabasal cells in the IFE, the epidermal proliferative units (EPU) hypothesis was widely accepted before quantitative lineage tracing experiments. This model suggested that a central slowly-cycling stem cell gives rise to TA cells, which divide a few times to maintain a column of differentiated keratinocytes, forming a columnar clonal unit [1,11,12].

Recent data from quantitative lineage tracing experiments, however, supported the idea that homeostasis is ensured by an “equivalency model” [9]. According to this model, the IFE is maintained by a homogeneous cell population, the CP cells, which proliferate equally at an average rate to produce proliferative and differentiated progeny in a stochastic manner [6–8]. In summary, the IFE undergoes constant turnover requiring proliferating progenitor cells, while HFSCs can remain quiescent and are only periodically required to maintain cyclical hair growth.

**Figure 1:**
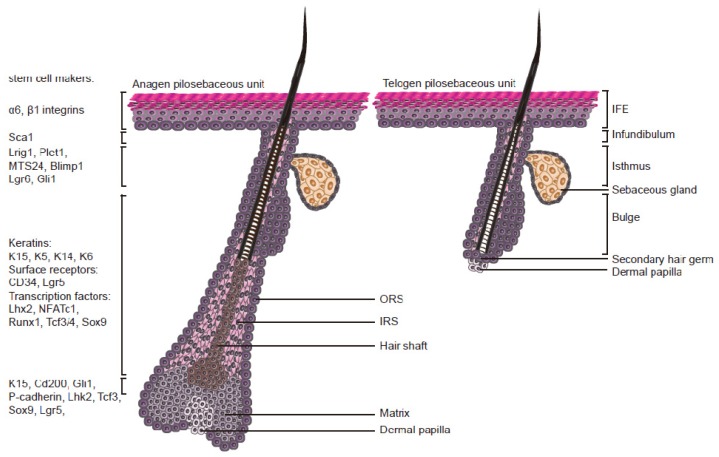
Schematic illustration of the multiple stem cell populations residing in the anagen and telogen pilosebaceous unit. Cells in the IFE express α6 β1 integrins. The infundibulum and the isthmus contain stem cell antigen-1 (Sca1), leucine-rich repeats and immunoglobulin-like domains1 (Lrig1), placenta-expressed transcript 1 (Plet1), MTS24, B lymphocyte-induced maturation protein 1 (Blimp1), Leu-rich repeat-containing G protein-coupled receptor 5 (Lgr5) and Gli-expressing cells which have an important role in wound healing [14–19]. The bulge contains a heterogeneous stem cell population. The outer layer of the bulge contains specific markers responsible for stemness maintenance, such as cell surface proteins (CD34 and Leu-rich repeat-containing G protein-coupled receptor 5 (Lgr5), keratins (K5, K14, K15) and transcription factors (Sox9, nuclear factor of activated T cells cytoplasmic 1 (NFATc1), LIM homeobox 2 (Lhx2), T cell factor 3 and 4 (Tcf3, Tcf4), Runx1). The inner layer of the bulge forms later during the hair cycle and responsible for maintaining HFSCs quiescence. It expresses K6 and some of the above-mentioned HFSC markers (Sox9, NFATc1, Lhx2 and Tcf3) [20–29]. The base of the bulge, the secondary hair germ, expresses placental cadherin (P-cadherin), Lhx2, Tcf3, Cd200, Gli1 and K15. However, many classical bulge markers are either absent (CD34 and NFATc1) or expressed at low levels (Lgr5 and Sox9) [21,30].

Hair follicles, appendages of the mammalian epidermis, are present all over the skin except the palms and eyelids. During embryonic development, hair follicle development starts with thickening of the epithelium to form a placode, which is closely associated with the underlying mesenchymal dermal condensation (DC); both structures are common to other ectodermal appendages, including feathers, teeth and mammary glands. The hair follicle is composed of concentric rings of external outer root sheath (ORS) cells attached to the basal membrane, a channel padded by the inner root sheath (IRS) cells and the hair shaft. The base or the bulb of the hair follicle contains the matrix cells, the actively proliferating TA cells and a cluster of specialized mesenchymal cells, the dermal papilla (DP) [13] (Figure 1).

Pulse-chase experiments have shown that the vast majority of slow-cycling, label-retaining cells (LRCs) are located in a special niche, the bulge, which is a well-protected, highly vascularized and innervated area [3,20,31]. It is located at the attachment site of the arrector pili muscle, below the opening of the sebaceous gland at the lower end of the non-cycling part of the hair follicle [3] (Figure 1). Besides stem cells, the bulge niche includes the inner bulge cells, DP, adipocyte precursor cells, subcutaneous fat and dermal fibroblasts, which provide important signaling cues for the residing stem cells [32].

### 2.3. The Hair Cycle

**Figure 2:**
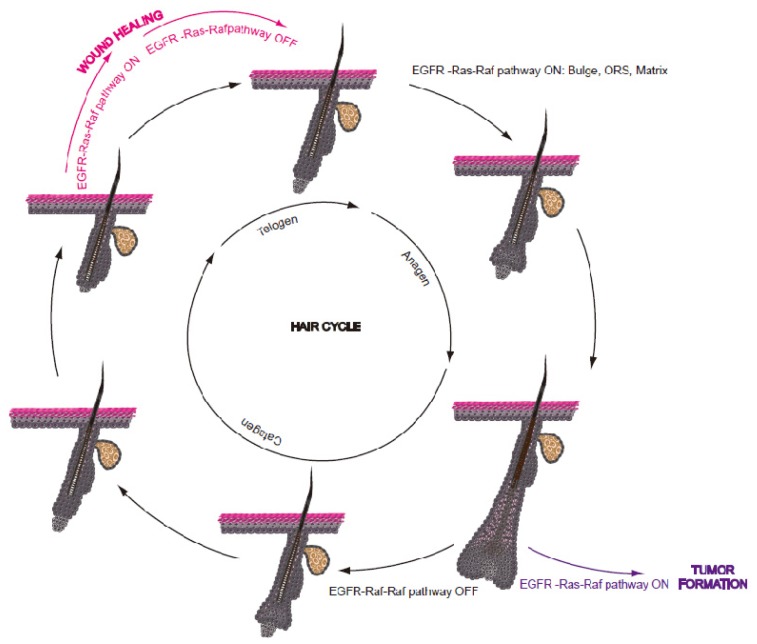
The EGFR-Ras-Raf pathway in hair follicle development and integrity. The hair cycle consists of several phases, namely anagen, catagen, and telogen. In physiological conditions, cyclical on/off switching of the EGFR-Ras-Raf pathway is required for hair cycle progression. Following injury, this cyclical rhythm is temporarily perturbed until the wound is repaired; continuous pathway activation, however, can lead to tumor formation.

HFSCs are quiescent during most of the hair cycle [3,20]. During the late telogen, the bulge is dormant, enriched in cell-cycle inhibitors and signaling repressors, generating an inhibitory niche. The two-step mechanism for stem cell activation involves first the secondary hair germ (sHG or hair germ-HG) interaction with DP, followed by the interaction between the sHG and the bulge [30,33–36]. The sHG, which is thought to be derived from the bulge [37,38], is a small cluster of P-cadherin-enriched cells that forms during telogen and separates the bulge from the underlying DP [39]. On a molecular level, stem cell activation depends on two critical pathways, the activation of the WNT pathway and the inhibition of the BMP signaling, which together lead to β-catenin stabilization [14,30,40–42]. During initiation of the anagen phase, activated HFSCs proliferate, exit the bulge and migrate downward along the ORS, pushing in front the DP. As the distance between the two increases, the DP can no longer activate the bulge, which returns to quiescence. However, DP can still activate the lower ORS, which proliferates and generates TA matrix cells. These cells form a bulb-like structure around the DP and further differentiate to from the IRS and the hair shaft [5,21,24,43,44]. After a limited number of cell divisions, catagen ensues: the matrix cells and the lower ORS undergo apoposis and the remaining epithelial strand pulls back the DP, whereas the residual upper ORS create the new outer bulge layer and sHG [37,38]. The K6^+^ inner bulge cells, which originate from the apoptosis-resistant lower ORS cells, express quiescence-inducing factors ensuring stem cell dormancy during telogen [28]. They are also responsible for anchoring the hair club during telogen and they can be removed upon hair plucking, leading to plucking-induced hair cycle activation [28]. Beside K6+ inner bulge cells, the dermal fibroblasts and subcutaneous adipocytes ensure the quiescent state of the telogen niche by the expression of BMP2 and 4 [45]. Although the lengths of murine anagen and catagen phases are comparable in each hair cycle, the telogen periods become progressively longer with age [2] (Figure 2).

## 3. The Role of EGFR-Ras-Raf Pathway in the Pilosebaceous Unit

### 3.1. The EGFR-Ras-Raf Pathway

The EGFR family comprises four members, EGFR/ErbB1/HER1, ErbB2/HER2, ErbB3/HER3 and ErbB4/HER4. As ErbB2 is an orphan receptor that still can be an EGFR co-receptor and ErbB3 and ErbB4 are either less active or have no known role in the epidermis, EGFR seems to play the main role in epidermal homeostasis and hair growth [46–48]. Besides EGF, EGFR can bind several other activating ligands, including transforming growth factor-α (TGFα), amphiregulin (AR) and epigen (EPGN), which can exclusively bind to EGFR. Heparin-binding EGF-like growth factor (HBEGF), betacellulin (BTC), epiregulin (EPR, EREG) can activate both EGFR and ErbB4 receptors. The ligands can be shed from a membrane bound precursor and bind the receptor via an autocrine, paracrine, or endocrine way; alternatively, they can remain membrane-bound and signal in a juxtacrine manner [49]. Upon ligand binding, receptor homo- or heterodimers are formed, leading to auto- and transphosphorylation events and receptor activation.

Besides activating the phosphoinositide-3 kinases (PI-3K)/Akt, JAK/STAT or PLCγ/PKC pathways, EGFR downstream signaling can progress through the Ras-Raf-mitogen-activated protein kinase (MAPK) signaling cascade, which is one of the best characterized effector pathways [50–54]. Briefly, activated EGFR receptor binds the docking protein Grb2 and the guanine nucleotide exchange factor SOS, which in turn activates Ras (H-Ras, K-Ras, N-Ras) by exchanging GDP for GTP. By binding and activating Raf (A-Raf, B-Raf, C-Raf/Raf-1), GTP-bound Ras launches the three-tiered kinase cascade, which is one of its key downstream effector pathways. In particular, K-Ras has been reported to have a preference towards Raf, while H-Ras favors PI3K activation [55]. Active Raf phosphorylates and activates MEK1/2, which in turn phosphorylates and activates ERK1/2. Activated ERK phosphorylates over 160 cytoplasmic and nuclear proteins, such as cytoskeletal proteins, kinases, phosphatases and transcription factors [56].

All Rafs can bind and phosphorylate MEK, but B-Raf has the strongest activity towards MEK, while A-Raf and Raf-1 have MEK-independent functions [50,52]. The kinase-independent function of Raf-1 is well demonstrated in the epidermis, where activation of ERK is not affected by Raf-1 ablation and Raf-1 can physically interact with, and inhibit, Rok-α [57–59]. Rok-α phosphorylates and activates LIM kinase, which in turn phosphorylates and inactivates cofilin. Besides its actin-depolymerizing activity, active (unphosphorylated) cofilin can enhance the phosphorylation of the transcription factor STAT3 and the expression of its target gene c-*myc* [58,60]. Taken together, Raf-1’s function as an endogenous Rok-α inhibitor is necessary for Stat3 and Myc activation [57–59,61].

### 3.2. The Effect of EGFR-Ras-Raf Pathway Deregulation on HF Integrity

The 80-year long investigation on EGFR signaling in skin homeostasis started with the description of the wavy hair phenotype in mice with loss of function of transformed growth factor alpha (TGFα) or EGFR [62,63].

EGFR is mainly expressed in the ORS, in basal and, to a lesser extent, in suprabasal keratinocytes [63,64]. EGFR activity maintains proliferation levels of the basal layer [65,66]. In turn, in the suprabasal layers, EGFR inhibition causes differentiation of the keratinocytes, which is associated with the induction of differentiation markers, such as K1 and K10 [65,67–70]. Consistent with this, activation of the EGFR downstream components Ras-Raf-ERK has been associated with increased proliferation and decreased differentiation [71–74].

The EGFR ligands have overlapping functions and expression patterns as demonstrated by the lack of skin abnormalities have been detected in mice deficient in EGF, AREG, or BTC [47]. EGF, which together with TGFα is the best-characterized EGFR ligand, is expressed in differentiating keratinocytes, sebocytes, and in the ORS, while TGF-α is expressed in the basal and differentiating layers of the epidermis and in the IRS [75–78].

Detailed analysis of the role of EGFR in hair follicle cycling has been impaired by the early mortality of the EGFR knockout mice. EGFR deletion or transgenic expression of a dominant-negative EGFR is lethal during embryonic development, but certain strains of mutant mice, which can survive several weeks, have severe skin abnormalities including epidermal atrophy, low epidermal keratinocyte proliferation rates, failure to develop a hairy coat or progressive alopecia and premature hair follicle differentiation [79–85]. Grafting EGFR-deficient skin on athymic nude mice revealed that *Egfr*^−/−^ follicles are proliferative, but differentiate prematurely and are not able to proceed from the anagen to catagen phase of the hair cycle, leading to necrosis and inflammation [86]. These data suggest that EGFR signaling protects hair follicles from autoreactive inflammation. Furthermore, decreased EGFR activity caused side effects like trichomegaly, cutaneous inflammatory rash, elongation, and wavy appearance of the scalp in patients treated with either anti-EGFR monoclonal antibodies (cetuximab, panitumumab) or small-molecule EGFR tyrosine kinase inhibitors (gefitinib, erlotinib), which are in clinical use for the treatment of metastatic epithelial cancers [87,88].

Although no obvious skin phenotype is present in *egfr*^+/−^ mice, further decrease in EGFR-Ras-Raf pathway activity causes milder phenotypes than EGFR deficiency, manifesting as delay of hair follicle development, disorderly oriented hair follicles, wavy coat and curly whiskers. In general, hair follicles are unable to exit the anagen and to enter catagen, leading to progressive hair loss and inflammation (see Table 1 for detailed descriptions of the mutants).

In contrast, increased EGFR-Ras-Raf pathway activity delays or blocks development of the murine hair follicles, at the final stage, reduces the hair diameter and increases proliferation in the basal layer [75,98,99]. In line with this, *in vitro* studies with isolated human scalp hair follicles have shown that EGF-induced proliferation in the ORS resulted in hair follicle elongation without hair growth. Interestingly, the matrix cells were produced in excess and squeezed up into the place of the hair club without entering apoptosis, suggesting that EGFR signaling might also be a stem cell activator during anagen induction [107].

Germline activating mutations in the Ras-Raf-MAPK pathway, referred as RAS/MAPK syndromes or RASopathies, are associated with cutaneous, cardiac, craniofacial defects and cancer predisposition. Three RAS/MAPK syndromes, Costello, CFC and Noonan syndrome, exhibit a wide range of ectodermal defects, including thickened palms and soles, redundant skin and papilloma formation [108]. Progressive hair and eyebrow loss, curly, poor hair growth is reported and alopecia is more frequent in CFC (59%) than in Costello (30%) patients, where *KRAS*, *BRAF*, *MEK1*/*2*, or *HRAS* can be mutated, respectively [109]. In Noonan patients, where *KRAS*, *PTPN11*, *RAF1* and *SOS1* mutation are frequent, curly hair is detected [110,111]. According to the mutational data from the Costello and CFC patients, it seems that Ras-Raf-MEK pathway has a significant role in regulating the hair cycle.

Mukhopadhyay and colleagues generated a mouse strain, which expresses physiological levels of an activated *KRas* (*KRas^G12D^*) allele along the midline epidermis and hair follicles (*Msx2*-cre; *Kras^G12D^*). The single *KRas^G12D^* allele induces proliferation in the basal keratinocytes, sebaceous gland and ORS, manifested in redundant skin folds, progressive hair loss, and spontaneous papillomas arising mainly on non-hair-bearing areas [105,106]. While *Kras* activation has a marked effect on the hair follicles, changes in downstream RAS/MAPK effectors were minor and only transcriptional changes, but no effects on cell signaling were observed. Surprisingly, even these small changes led to reduced levels of Sonic hedgehog (Shh), which controls hair follicle morphogenesis in embryonic skin and telogen-anagen transition in postnatal skin [106]. In line with this, Shh blockade results in the arrest of hair follicle morphogenesis or deregulates telogen–anagen transition [112–115]. Thus, the reported phenotypes of Ras gain-of-function mutations might partially result from decreased Hedgehog pathway activity.

In their earlier work the authors have shown that *Msx2*-cre; *Kras^G12D^*mice exhibit defective activation of hair cycle during the first postnatal hair cycle, even after trimming-induced anagen. The defect in the postnatal hair cycle activation is the consequence of overgrown ORS, sHG and of matrix cells that fail to undergo apoptosis during catagen [105]. Most likely, the unusual expansion of the ORS cells during the hair follicle development pushes the sHG and DP away from the bulge to a distance from where their paracrine signal from the DP cannot activate the bulge cells and initiate anagen.

Epidermis-restricted B-Raf or Raf-1 knockout mice exhibit delayed hair follicle development. In addition, Raf-1 ablation causes defects in keratinocyte migration, wound healing, and mild waviness of the fur, which disappears after the first hair cycle [57,58]. Interestingly, mice with double knockout B-Raf and Raf-1 epidermis show curly, sparse hair and progressive hair loss, indicating that B- and Raf-1 have overlapping functions regulating the hair cycle [116].

Finally, inhibition of the Raf-1 interactor Rok-α promotes survival and inhibits the differentiation of human embryonic stem cells and human keratinocytes, suggesting a role in stem cell maintenance [58,60,117–119].

Collectively, these results indicate that EGFR-Ras-Raf signaling is absolutely required for the proper timing of the first onset of catagen, for hair cycle progression and for maintaining hair follicle integrity during the hair cycle (Figure 2).

## 4. The EGFR-Ras-Raf Pathway in Wound Healing

An accurate balance of proliferation and differentiation is required to control tissue homeostasis in the adult epidermis. This balance is shifted towards proliferation upon skin injury and must be set back after wound healing is completed.

Wound healing is a complex process involving a number of well-coordinated events such as inflammation, cell proliferation and migration, matrix production and angiogenesis. It starts with an acute inflammatory phase in which neutrophils and macrophages are recruited to the wounded site right after the injury. The formation of a fibrin clot serves as temporary repair unit and as a scaffold for infiltrating cells, followed by the establishment of granulation tissue produced by fibroblasts and macrophages. The closure of the wound is mediated by the contractile granulation tissue, which draws the wound edges closer together. Moreover, keratinocytes start to proliferate at the wound margin and migrate into the granulation tissue until re-epithelialization is complete [120].

During tissue homeostasis, the HFSCs have specific and distinct functions and do not contribute cells to the epidermis, they, however, can behave as multipotent stem cells during physical injury, and can participate in the wound re-epithelialization [121–123]. In contrast to the downward movement at the initiation of anagen, the bulge stem cells migrate upward during wound healing and stay several weeks in the newly formed epidermis [21,43,122]. Gli1, Lgr6 and Lrig1-expressing keratinocytes from the upper isthmus and the infundibulum contribute permanently to the epidermis and give rise to all epidermal cell lineages after wounding [15–17,24,122,124]. Following injury and until the cellular integrity has been fully restored, stem cells show increased proliferation and decreased differentiation levels [125].

The EGFR-Ras-Raf pathway has been implicated in the wound healing process. HBEGF is the most abundant growth factor in wound fluid; it is rapidly induced after injury and plays an important role in wound-induced keratinocyte migration and adhesion [126–128]. EGFR activation contributes to the proper timing of wound re-epithelialization by increasing keratinocyte proliferation, migration and angiogenesis and by mediating inflammation (Figure 2). EGFR activation is important, but not absolutely necessary for this process, as wound closure, albeit delayed, is also accomplished in the absence of EGFR [129].

The kinase-independent function of Raf-1 is required for keratinocyte and fibroblast migration *in vitro* and it is an important player in wound healing *in vivo*. Raf-1-deficient cells show impaired migration and abnormal cellular shape due to the hyperactivation and plasma membrane localization of Rok-α [57]. Tissue damage, inflammation, and cancer development are closely connected, the main difference being that wound healing is a self-limiting process while tumorigenesis is marked by constitutive pathway activation [125,130]. However, both processes use similar signaling pathways, including Ras, Hedgehog and WNT [131–133]. During both tissue repair and tumor development, stem cells are found outside of their usual niche, and the dynamic interaction between them and this changed microenvironment, containing mesenchymal, bone marrow-derived cells and immune cells, plays a crucial role in their activation [132].

## 5. The EGFR-Ras-Raf Pathway during Non-Melanoma Skin Carcinogenesis

### 5.1. Squamous Cell Carcinomas (SCC)

Cutaneous squamous cell carcinoma (cSCC) is the second most frequent human cancer, with more than 500,000 new cases annually worldwide [134]. It typically exhibits a broad spectrum of progressively advanced malignancies, ranging from premalignant actinic keratosis (AK) to squamous cell carcinoma *in situ* (SCCIS), invasive cSCC and finally metastatic cSCC. The primary risk factor for AK is chronic UV exposure [135], and the estimated rate for an individual lesion to progress to cSCC is between 0.025% and 16% per year [136]. In patients with metastatic cSCC, however, the prognosis is very poor, with only a 10%–20% survival rate over 10 years [134]. Histologically, AKs are characterized by dysplasia of the keratinocytes in the basal layer, often accompanied by parakeratosis and thinning of the granular layer. This localized epidermal atypia reflects a partial disruption of the differentiation program, whereas a more complete loss of differentiation is associated with cSCCs. Genetically, AKs and cSCCs are associated with amplifications and activating mutations of the Ras oncogene; the latest data from the catalog of somatic mutations in cancer (COSMIC; Sanger Institute, Hixton, Cambridgeshire, UK) indicate that 11% of cSCCs harbor activating Ras mutations (6% HRAS, 3% NRAS, 2% KRAS; *n* = 371 cases) [137]. In a three-dimensional organotypic model of human epidermis, it is sufficient to couple Ras overexpression with the activation of the cell cycle progression mediator CDK4, or to modulate NF-κB activity to bypass Ras-mediated G1 arrest to induce epidermal tumorigenesis [138–140]. This suggests that apart from rarely detected activating mutations, other factors, such as the overexpression of receptor tyrosine kinases upstream of Ras, might activate the pathway in tumors [141].

Increased cSCC formation in patients is associated with medical conditions and drug usage in several clinical situations [140]. Most prominently, treatment of BRAFV600E mutated melanoma with the ATP competitive kinase inhibitors vemurafenib (PLX4032) or dabrafenib (GSK2118436) leads to the development of keratoacanthomas or cSCCs in up to 30% of the patients [142–145]. These Raf inhibitors paradoxically stimulate Raf kinase activity by promoting dimerization and kinase maturation particularly in the presence of activated Ras. In support of this, Raf inhibitors accelerate tumor development in a mouse model of chemical skin carcinogenesis that induces activating mutation of H-Ras [53,144,146–149].

Chemically induced carcinogenesis is the most commonly used cSCC model in mice [150,151]. This protocol consists in the single administration of an “initiating” carcinogen (7,12-dimethylbenz-alpha-anthracene; DMBA) followed by chronic treatment with a “promoting” agent (12-*O*-tetradecanoyl-phorbol-13-acetate; TPA). During the course of TPA administration, benign tumors (papilloma) arise, which then at low frequency progress to invasive cSCCs. The period between the initial DMBA treatment and the subsequent TPA application can be extended for up to one year without significant loss of effectiveness of promoter treatments, suggesting that the initial HRas mutation arises in long-lived stem cells [152]. To dissect the cellular origin of cSCCs in more detail, oncogenic *KRas^G12D^* was expressed in several compartments of the hair follicle by two independent groups [153,154]. Expression of mutant *KRas* in hair follicle bulge stem cells and their immediate progeny (hair germ and outer root sheath), but not in the transient amplifying matrix cells, led to the development of benign squamous skin tumors. Whereas only benign tumors were observed after *KRas^G12D^* expression alone, combined p53 deletion and oncogenic *KRas* expression initiated invasive cSCCs. Consistent with this finding, around 40% of human cSCCs harbor p53 mutations (COSMIC; Sanger Institute, Hixton, Cambridgeshire, UK), indicating that p53 loss might be tightly associated with cSCC progression [155]. The decreased incidence of tumor formation observed in the DMBA/TPA model after removal of the IFE by dermoabrasion suggested that cSCCs may not only arise from the hair follicles but also from the IFE [156]. Indeed, IFE progenitors were able to form papillomas following mutant *KRas* or *HRas* expression [153,157]. In contrast to tumors induced in suprabasal, more differentiated layers of the epidermis, the formation of these lesions was not depending on wounding [157,158]. In summary, these studies showed that the ability of oncogenic Ras to induce skin tumors is very much dependent on the cell type targeted.

In a recent study, Malanchi *et al.* identified a population of cells in DMBA/TPA derived SCCs with phenotypic and functional properties similar to those of normal bulge skin stem cells [159]. These cells specifically expressed CD34 and other established markers of bulge skin stem cells, and after transplantation efficiently initiated secondary tumors that recapitulated the organization of the primary tumors. Ablation of β-catenin resulted in loss of the cancer stem cell properties and tumor regression. Interestingly, benign papillomas formed secondary tumors upon transplantation at very low frequency, and only when tumor cells were co-transplanted together with tumor-associated fibroblasts or endothelial cells. The frequency of tumor propagating cells generally increased with tumor progression [160].

Whereas in these initial studies cancer stem cell properties have been investigated by transplantation assays, the Blanpain lab has recently demonstrated the existence of tumor cells with stem-cell-like properties in cSCCs within their natural microenviroment [161]. Using a genetic labeling strategy that allows individual tumor cells to be marked and traced over time at different stages of tumor progression they could show that the majority of tumor cells in benign tumors has only limited proliferative potential, whereas a fraction of cells with stem-cell-like characteristics has the capacity to persist long term and to give rise to progeny that makes up most of the tumor. The presence of two distinct proliferative compartments within the tumor closely mirrors the composition, hierarchy and cell fate behavior of normal tissue. In contrast to papillomas, most cells in invasive cSCCs were found to be proliferative with no signs of terminal differentiation.

### 5.2. Basal Cell Carcinoma (BCC)

Basal cell carcinoma is currently the most common human cancer in several countries [162]. The tumors are locally invasive but very rarely metastatic and their incidence is closely associated with UV exposure. BCCs display a broad variety of growth patterns and are characterized by a primary cellular component that resembles the undifferentiated basal cells of the epidermis and its appendages [163]. Genetically, the vast majority of sporadic BCCs arise from mutations that constitutively activate the Hedgehog (HH) pathway [164]. The family of extracellular HH ligands (SHH, IHH and DHH in mammals) bind to the patched 1 receptor (PTCH1) which relieves the inhibition of smoothened (SMO) by PTCH1, resulting in activation of the downstream Gli family of transcription factors (GLI1-3). BCCs either arise through PTCH1 loss of function or by activating mutations in the *SMO* gene. In 2012 the FDA approved vismodegib, a SMO antagonist that competes with the natural inhibitor cyclopamin, for their treatment [165].

Several recent studies describe the use of genetic mouse models to study the cellular origin of BCCs either by lineage tracing or by the activation of oncogenic HH signaling in distinct cell populations [163,166]. Specifically, in mice conditionally expressing a constitutively active variant of SMO (SmoM2), spontaneous BCC arises from long-term resident progenitor cells of the IFE and the upper infundibulum [167], while hair follicle bulge stem cells and their transient amplifying progenies do not induce cancer formation unless recruited to wound sites [168]. Wounding also increases the frequency of BCCs induced by homozygous Patched loss of function [169], but the initiating cells in this case are reportedly hair follicular stem cells [170]. In the SmoM2 tumors, the initiating IFE progenitor cells are massively reprogrammed into a fate resembling that of embryonic hair follicle progenitors, indicating that the expression of differentiation markers by tumor cells can be misleading when used to identify their cellular origin [171]. One of the earliest molecular changes during this reprogramming process includes the activation of Wnt/β-catenin signaling, on which the development of BCCs critically depends [172]. Eberl *et al.* recently demonstrated that SmoM2-driven BCC formation depends not only on WNT but also on EGFR signaling, as epidermal- specific deletion or pharmacological inhibition of EGFR reduced both the number and size of tumors [173]. A screen for downstream mediators identified a number of HH-EGFR cooperation response genes, including the transcription factors Sox2 and Sox9, involved in the regulation of stem cell fate, or FGF19 and CXCR4, which might be exploited for novel therapeutic approaches. Mechanistically, the expression of these cooperation response genes was suggested to be regulated at the transcriptional level by cooperative interactions of the GLI activator forms (GLI-A) and JUN/AP1 transcription factor downstream of the RAS/RAF/MEK/ERK cascade [174].

## 6. Conclusions

80 years of study on the EGFR-Ras-Raf pathway have broadened our knowledge about its functions in different organs during homeostatic and pathologic conditions, and have provided a basis for cancer therapies. Decrease in the EGFR pathway activity leads to changes in the morphology and distribution of hair follicles, manifested in wavy coat and curly whiskers [2,62,63,83–85,89,92,94,175]. However, continuously activated signaling in most of the cases leads to hair loss, supporting the theory that a cyclical on/off switch of this pathway is required for proper hair cycle progression [75,98,99,105–107] (Table 1). Thus, the epidermis and its appendages react in a very sensitive, easily monitorable manner to the modulation of the EGFR-Ras-Raf pathway and would be an excellent system to study the effect of this pathway on somatic stem cells. Several open questions can now be addressed by combining conditional gene ablation with the use specific epidermal stem cell markers and quantitative lineage tracing. For instance, in which epidermal stem cell population is the EGFR-Ras-Raf pathway active? How does it affect the bulge and non-bulge stem cells during wound healing? How is the pathway turned on during anagen induced stem cell activation and off at the end of anagen? In addition, in tumors, what is the influence of the stroma on the growth properties of cancer stem cells? How is the balance between proliferation and differentiation regulated during tumor progression? Does the cellular reprogramming event following oncogene expression in BCCs play a causal role during tumor initiation and what is the contribution of the EGFR-Ras-Raf pathway to it?

With the increasing clinical use of EGFR monoclonal antibodies and EGFR and Raf inhibitors, future studies should concentrate on better understanding of the EGFR-Ras-Raf signaling in different epidermal stem populations during hair morphogenesis, hair cycle and pathologic conditions.

## Figures and Tables

**Table 1:** Abnormalities in mouse hair follicle morphogenesis due to dysregulated EGFR-Ras-Raf pathway.

Model	Phenotypes	References
**Hypomorphic phenotypes**		
Epidermal growth factor receptor (EGFR)^−/−^	Open eyes, rudimentary whiskers, defects in epidermis, delay of hair follicle development, disoriented hair follicles	[79–81]

Epidermis restricted dominant negative mutant of EGFR	Short and waved pelage hair, curly whiskers, hairs fail to enter catagen, thinning or loss of the ORS and IRS	[82]

Epidermis specific deletion of EGFR exon 3 (K14-Cre, EGFR^tmDwt^), (abrogated ligand binding)	Wavy coat hair, curly whiskers	[83]

Humanized conditional EGFR knock-in (*hEGFR^KI/KI^*) (the new allele is not efficiently expressed in the skin)	Homozygotes exhibit skin and hair defects similar to Egfr^−/−^ mutants leading to progressive hair loss	[89]

Point mutation (Val 743 Gly) in the EGFR kinase domain (waved-2 allele)	Phenotype of the homozygotes are similar to TGF alpha^−/−^	[63,90]

EGFR waved-2 (see above) Ptpn11^+/−^ (Protein tyrosine phosphatase, non-receptor type11)	Few poorly developed and disordered hair follicles	[91]

Dominant negative (Asp 833 Gly) mutation in the EGFR DFG motif in the kinase domain (waved-5/velvet allele)	Heterozygous mice have open eyes and wavy coat and curly whiskers. Homozygous mice die at midgestation owing to placental defects	[84,85]

Transforming growth factor-α (TGFα)^−/−^ (or spontaneous TGFα waved 1 mutation)	Wavy coat hair, curly whiskers	[62,92]

AR^−/−^; EGF^−/−^; TGFα^−/−^	Wavy coat hair, curly whiskers	[93]

A Disintegrin and Metalloproteinase 17 (ADAM17 ) deletion in keratinocytes (A17(ΔKC)(responsible for the TGFα shedding)	Defects in epidermal barrier integrity, chronic dermatitis	[94]

Ksr1^−/−^ (Kinase suppressor of Ras1) (positive scaffolding modulator of Ras/MAPK signaling)	Short wavy hair, progressive hair loss, disorganized hair follicles, asynchronous hair growth, IRS separated from the hair shaft	[95]

Mek1 (Mitogen activated protein kinase kinase 1)^−/−^	Reduced hair follicle proliferation	[96]

**Hypermorphic phenotypes**		

Missense mutation ( Leu863Gln ) in the EGFR kinase domain (Dsk5 allele)	Wavy coat, curly vibrissae that becomes less apparent with age and thickened, hyperpigmented epidermis	[97]

Continuous expression of EGF in hair follicles	Retarded hair follicle development, reduced hair diameter and increased proliferation in the basal layer	[75,98,99]

Skin-specific overexpression of TGFα	Diffuse alopecia, hyperkeratosis, spontaneous SCC development, wrinkled skin	[100]

K14 driven TGFα expression	Low hair follicle density, distorted hair follicles, reduced hair growth and thick epidermis	[101]

Skin-specific overexpression of human amphiregulin (AR)	Psoriasis-like skin phenotype, alopecia	[102]

Ubiquitous overexpression of betacellulin (BTC)	Waved coat and delayed hair follicle morphogenesis and hair cycle induction	[103]

Ubiquitous overexpression of human epigen (EPGN)	Enlargement and hyperactivity of the sebaceous glands	[104]

Activated Kirsten rat sarcoma viral oncogene homolog (KRasG12D) expression in the midline epidermis	Defective anagen entry, progressive hair loss, overgrown ORS, sHG and matrix cells failing to undergo apoptosis	[105,106]
